# The impact of hypocrisy on opinion formation: A dynamic model

**DOI:** 10.1371/journal.pone.0218729

**Published:** 2019-06-26

**Authors:** Michael T. Gastner, Károly Takács, Máté Gulyás, Zsuzsanna Szvetelszky, Beáta Oborny

**Affiliations:** 1 Division of Science, Yale-NUS College, Singapore, Singapore; 2 MTA TK “Lendület” Research Center for Educational and Network Studies (RECENS), Hungarian Academy of Sciences, Budapest, Hungary; 3 The Institute for Analytical Sociology (IAS), Linköping University, Norrköping, Sweden; 4 Department of Plant Taxonomy, Ecology and Theoretical Biology, Biological Institute, Loránd Eötvös University (ELTE), Budapest, Hungary; 5 GINOP Sustainable Ecosystems Group, Centre for Ecological Research, Hungarian Academy of Sciences, Tihany, Hungary; IT University of Copenhagen, DENMARK

## Abstract

Humans have a demonstrated tendency to copy or imitate the behavior and attitude of others and actively influence each other’s opinions. In plenty of empirical contexts, publicly revealed opinions are not necessarily in line with internal opinions, causing complex social influence dynamics. We study to what extent hypocrisy is sustained during opinion formation and how hidden opinions change the convergence to consensus in a group. We build and analyze a modified version of the voter model with hypocrisy in a complete graph with a neutral competition between two alternatives. We compare the process from various initial conditions, varying the proportions between the two opinions in the external (revealed) and internal (hidden) layer. According to our results, hypocrisy always prolongs the time needed for reaching a consensus. In a complete graph, this time span increases linearly with group size. We find that the group-level opinion emerges in two steps: (1) a fast and directional process, during which the number of the two kinds of hypocrites equalizes; and (2) a slower, random drift of opinions. During stage (2), the ratio of opinions in the external layer is approximately equal to the ratio in the internal layer; that is, the hidden opinions do not differ significantly from the revealed ones at the group level. We furthermore find that the initial abundances of opinions, but not the initial prevalence of hypocrisy, predicts the mean consensus time and determines the opinions’ probabilities of winning. These insights highlight the unimportance of hypocrisy in consensus formation under neutral conditions. Our results have important societal implications in relation to hidden voter preferences in polls and improve our understanding of opinion formation in a more realistic setting than that of conventional voter models.

## 1 Introduction

Public opinion is formed as a result of interrelated changes in individual opinions. Through a large number of social interactions, humans are greatly influenced by the opinions, attitudes, and behavior of others. The dynamics resulting from these interactions have been studied in various models [1–9]. One of the most appealing and widely studied representations is the voter model due to its simplicity [10–14]. The paradigmatic voter model (which we will call the Basic Voter Model, BVM) represents the opinions of individuals as a binary variable in a single opinion dimension. At each update, an individual adopts the opinion of one of his acquaintances. In the BVM, every individual is assumed to express his inner conviction.

In practice, however, there might be a difference between an individual’s publicly revealed and internal opinion. In the present paper, we focus on *hypocrisy* (i.e., individuals exhibit “self-censored” opinions in public [15]) and the abundance of *hypocrites* (i.e., individuals whose external and internal opinions differ). We study their frequency in the group and their role in the consensus-finding process.

Hypocrisy is abundant in all aspects of human social life. Voters withhold their true opinions in opinion polls, causing difficulties for the prediction of true outcomes [16–19]. Humans adjust their views and opinions to the circle in which they are present [20]. Social desirability is a general driver of expressing an opinion [21–23]. Even in the absence of direct influence or persuasion, individuals adjust their revealed opinion if they are exposed to the attitudes of others [24]. Actual behavior can fall very far from self-reports that are subject to social desirability bias, for instance in dietary intake [25], household labor [26,27], or physical activity [28]. Asch's classic experiments [29], in which he studied the influence of group pressure on individual opinions, show significant levels of conformity: participants of the experiments adopted the group's false opinion in a third of the trials, and more than half of the participants adopted a false group consensus at least once. Haun and Tomasello [30] demonstrated that, already at a young age, children try to say what they are expected to say without changing their own “real” judgment of the situation.

Many people attend church services without true faith, express popular opinions in discussions and meetings, follow a fashion trend they do not fancy, or adhere to regulations they disagree with. They show off with conspicuous consumption or by mowing their lawn while they have better things to do. People support morals they are not engaged with privately, have flexible virtues, and, following others, easily absolve themselves of moral responsibility [31–33].

Individuals might even openly support norms or legitimize a political system they privately do not sympathize with [34,35]. For example, Jiang and Yang [36] report quantitative evidence for “preference falsification” after a political purge in Shanghai in 2006. Laboratory experiments and computational models show that humans might punish norm violators in order to conform to group pressure against their own inner conviction [37,38]. The willingness to conform to social expectations might even lead to self-destructive behaviors such as binge drinking, shoplifting, and smoking among adolescents [39]. In extreme cases, this kind of hypocrisy can support a witch hunt, ostracism, or the public condemnation of scapegoats.

Hypocrisy is also a general feature of organizational behavior where reputation and promotions are at stake [40–42]. Within organizations, language use is often adjusted to the audience. For instance, the tone of political correctness depends on the expectations of receivers [43]. According to Noelle-Neumann [44], a significant proportion of people try to avoid isolation in the case of public questions. She posits that social relations and acceptance in their own environment are more important to people than revealing their own views in public.

All instances of hypocrisy create cognitive dissonance [45], which can be reduced mainly in two ways: internalization or externalization. In the case of internalization, the individual accepts the belief or opinion that he has expressed publicly [46]. Adjusting the internal opinion is an important last step in the process of socialization that is well described in the classic sociological and social psychological literature [47,48]. Opinions and individual attitudes are the products of contact with other members of the group that creates an internal and external conflict “with its resolution based on the internalization of external norms” [49]. Internalization is distinct from compliance as it covers the private acceptance of the norm or attitude [46]. As a result, the individual can be said to be depersonalized and fully assimilated to the group [50,51].

In the case of externalization, the previously concealed opinion becomes publicly expressed. In practice, this requires courage or expressiveness. The “coming out” necessarily results in public discomfort, but often produces relief, a positive cognitive and emotional state. Acting and producing a poker face for the inhibition of inner opinions is certainly costly [52], and the individual is liberated from these costs by expressing his internal belief. It depends on the empirical context to which extent it is feasible to reduce cognitive dissonance by internalization or externalization. For instance, externalization is particularly problematic for political opinions in oppressive regimes, but strongly encouraged when diverse standpoints need to be revealed such as in critical academic debates.

The objective of this article is to analyze the role that hypocrisy, internalization and externalization play in opinion formation. As a more realistic alternative to the Basic Voter Model (BVM), we study the Concealed Voter Model (CVM), in which the publicly expressed (external) opinion can differ from the internal one [53]. Before we give a detailed definition of both models in **Section 2**, let us first state and motivate their underlying assumptions. The BVM and CVM share the following simplifications:

- There are only two kinds of potential opinions (which we call “red” and “blue”) on a particular issue, and these are mutually exclusive.
- In the external layer, individuals interact in pairs.
- The group is homogeneous in the sense that each rate is the same for all individuals and at all times.
- Red and blue opinions have the same transition rates. That is, their competition is neutral.

**Fig 1:**
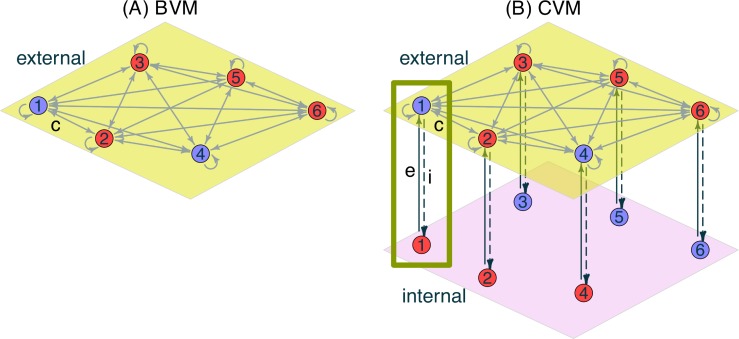
The Basic Voter Model (BVM) versus the Concealed Voter Model (CVM). ***(A)*** In the BVM, every individual is represented by a node in a single layer, which is “external” in the sense that all of its neighbors can see and copy his opinion. The tension that is caused by the disagreement between neighbors (for example between the blue individual 1 and the red individual 2) can be released by adopting the neighbor’s opinion. Such copies happen with a rate *c*. ***(B)*** In the CVM, every individual is represented by two nodes: one in the external and another in the internal layer. For example, let us consider individual 1, outlined by the green rectangle. His external/internal opinion is marked by a blue/red circle. Because these opinions differ, we call individual 1 a “hypocrite”. The tension between the external versus internal opinion can be released either by externalization or internalization (with the corresponding rates *e* and *i*). None of the individuals knows the internal opinion of any other individual. Hence, there are no edges within the internal layer.

Most versions of the voter model in the literature apply these simplifying assumptions (for reviews, see [12,14]). Extensions have relaxed some of the assumptions. For example, (A) was relaxed in the model by Vazquez and Redner [54], and (B) by Lambiotte and Redner [55]. Assumption (C) was relaxed by introducing zealots who never change their opinion [56,57] and (D) was relaxed in the biased voter model [58]. The CVM keeps (A)-(D) and adds an internal layer [53] (**Fig 1**). Some previous versions of the voter model have also considered a duality of opinions similar to our juxtaposition of external versus internal layer (e.g., [59,60]), but the internal opinions were static. A model that distinguishes between private and expressed opinions, but assumes continuous instead of binary opinions, was introduced by Ye et al. [61] and by Huang and Wen [62]. The CVM is the first discrete-state voter model in which the opinions in the internal layer can vary in response to the external layer within the individual, but not as a result of links to other internal opinions. In other multilayer voter models, nodes are connected in a multiplex network whose layers contain the same set of nodes with partially overlapping edges [63–67]. However, each layer in these multiplex models contains intra-layer edges so that none of the layers plays the role that the internal layer plays in the CVM: it contains private, hidden opinions that are unknown to other individuals.

Real-life examples for assumption (A) include those issues that are at stake in a social group and whose answer must be one of exactly two possibilities (e.g., yes/no or false/true). Many US elections, for instance, come down to a choice between only the Democrats or the Republicans. A recent example with exactly two alternatives on the ballot was the 2016 Brexit referendum, where voters could only choose between “remain” or “leave”.

Assumption (B) implies that external opinions can spread only from person to person so that the role of mass media is negligible. Mass media tend to portray the majority opinion as socially desirable and, hence, may increase the number of hypocrites in the population. Issues that are less influenced by mass media comply more with (B), for example newly trending issues (such as new slang expressions or underground music), shameful issues (e.g., norms of toilet use), or topics that are only of local or personal importance and interest (e.g., issues of taste, norms or habits in a small group or community).

Assumption (C) ensures an equal opportunity structure for the individuals in our model. It excludes the possibility that some of them can react to social influence, externalize, or internalize at a different rate from others. Assumption (D) guarantees that the dynamics does not give an intrinsic bias to one of the opinions. A typical asymmetry in real opinion formation may arise, for example, when one of the opinions provides more benefits in economic or social terms for the individuals than the other. We wish to exclude this kind of bias in order to study the process of spreading in a pure form. Thus, the BVM and the CVM can be used as neutral references to which more complex models or real-life systems can be compared.

Our previous results in the CVM have shown that, on average, the processes of internalization and externalization delay the consensus [53] because a minority opinion may still linger in the internal layer even when it has temporarily disappeared in public. In this paper, pursuing this path further, we ask fundamental questions about the role of hidden opinions and hypocrisy in the consensus formation process. We examine the proportion of hypocrites in the group over time and their effect on the dynamics of consensus formation. Our goal is to reveal how hidden opinions influence the diversity of public opinions over time and how they shape the dynamics leading to eventual unanimity.

## 2 Methods

In the Basic Voter Model (BVM), the opinion of each individual is one node in a single, external network layer (**Fig 1A**). By contrast, the Concealed Voter Model (CVM) represents each individual by two nodes: one opinion in the external and another in the internal layer, which do not need to be identical (i.e., hypocrisy is possible). In the BVM and CVM, we model the external layer as a complete graph. We include a link from each node to itself. The models with and without the self-link are in fact merely reparameterized versions of each other (see S1 Appendix). We include the self-link because it simplifies the notation of the equation-based analysis in the S1 Appendix. While the external nodes are thus maximally connected with each other, every node in the internal layer is linked only to the external node representing the same individual (**Fig 1B**).

We call the alternative opinions “red” or “blue” and denote the opinions in the external versus internal layer by capital versus lower case letters (*R*: external red, *r*: internal red, *B*: external blue, *b*: internal blue). In the BVM, one letter is sufficient to characterize the state of every individual: *R* or *B*. We denote the fraction of agents in these states as *ρ*_*R*_ and *ρ*_*B*_ = 1−*ρ*_*R*_. The group has reached a consensus when *ρ*_*R*_ = 1 or *ρ*_*B*_ = 1. In the CVM, we need two letters to describe the state: *Rb* (externally red hypocrite), *Br* (externally blue hypocrite), *Rr* (red frank) or *Bb* (blue frank). We denote the fraction of individuals in these states by *ρ*_*Rb*_, *ρ*_*Br*_, *ρ*_*Rr*_, and *ρ*_*Bb*_ respectively. The state of the whole group can be described by the combination (*ρ*_*Rb*_, *ρ*_*Br*_, *ρ*_*Rr*_). We do not need to explicitly include *ρ*_*Bb*_ because it is uniquely determined by *ρ*_*Bb*_ = 1−*ρ*_*Rb*_−*ρ*_*Br*_−*ρ*_*Rr*_. The abundances of internal and external opinions can be represented by the 2×2 matrix shown in **Table 1**. The proportion of hypocrites is *ρ*_*Rb*_+*ρ*_*Br*_. The group is in a consensus when *ρ*_*Rr*_ = 1 or *ρ*_*Bb*_ = 1. That is, we define a consensus as a state in which only one of the opinions exists; the alternative opinion has completely disappeared even from the internal layer.

**Table 1:** *Contingency table showing the fraction of individuals with different combinations of external and internal opinions*.

		External opinion		
		*R*	*B*	sum
Internal opinion	*r*	*ρ*_*Rr*_	*ρ*_*Br*_	*ρ*_*r*_
	*b*	*ρ*_*Rb*_	*ρ*_*Bb*_	*ρ*_*b*_
	sum	*ρ*_*R*_	*ρ*_*B*_	1

For both models, the consensus time *T*_cons_ is defined as the duration between the initial state and the consensus. (Its synonyms are exit time, hitting time, or first-passage time; cf. [58,68]).

An agent-based Monte Carlo simulation of the BVM with *N* individuals proceeds according to the following algorithm.

1. *Initialization*: We initialize the opinions such that a given fraction *ρ*_*R*_ of the nodes are red. All other nodes are blue. We initialize time: *t*←0.
2. *Iteration*:
  - We choose a “focal” individual *f* uniformly at random from all of the *N* individuals.
  - We pick a neighbor *n* of the focal individual uniformly at random from all of its neighbors. (On a complete graph with self-links, all individuals are neighbors of each other so that *n* can be any individual in the group.)
  - *f* adopts *n*’s opinion. That is, if *f* and *n* have different opinions, we change *f*’s opinion to that of *n*. Otherwise the system remains unchanged.
  - We increment the time by a random number Δ*t* drawn from an exponential distribution with mean $\frac{1}{cN}$, where *c* is a positive number: *t*←*t*+Δ*t*. We can interpret *c* as the rate with which individuals copy opinions of any of their neighbors.
  - If all nodes have the same color (i.e., the individuals have reached a consensus), we set $T_{cons}^{\left(BVM\right)}\leftarrow t$ and terminate. Otherwise we go back to step (i).

Compared to the BVM, the CVM adds two options to step (ii): besides copying a neighbor’s external opinion, individuals may also externalize or internalize (**Fig 1B**) to reduce cognitive dissonance as described in **Section 1**. We model copying, externalization and internalization as independent Poisson processes with rates *c*, *e* and *i*, respectively, according to the following agent-based algorithm.

1. *Initialization*: We initialize the opinions so that
  - a given fraction *ρ*_*Rb*_ of the individuals is externally red and internally blue,
  - a given fraction *ρ*_*Br*_ is externally blue and internally red,
  - a given fraction *ρ*_*Rr*_ is red in both layers,
  - the rest is blue in both layers.
    We also initialize time: *t*←0.
2. *Iteration*:
  - We choose a “focal” individual *f* uniformly at random from all of the *N* individuals.
  - We generate a random number *u* that is uniformly distributed between 0 and *c*+*e*+*i*. We now distinguish between three cases.
    *Case (A)*: *Copying*. If *u*<*c*, then *f* adopts the opinion of a random neighbor and we immediately go to step (iii).
    *Case (B)*: *Externalization*. Otherwise, if *c*≤*u*<*c*+*e*, then *f* externalizes. That is, if the current state of *f* is *Rb*, we change its state to *Bb*. If the current state is *Br*, we change *f*’s state to *Rr*. For all other current states, the system does not change. We subsequently go immediately to step (iii).
    *Case (C)*: *Internalization*. Otherwise *f* internalizes. That is, if the current state of *f* is *Rb*, we change its state to *Rr*. If the current state is *Br*, we change *f*’s state to *Bb*. In all other cases, the system does not change.
  - We increment the time (i.e., *t*←*t*+Δ*t*) by a random number Δ*t* drawn from an exponential distribution with mean $\frac{1}{(c+e+i)N}$. We can interpret *c*+*e*+*i* as the rate with which individuals are active because they copy, externalize, or internalize.
  - If all nodes have the same color (i.e., all internal and external opinions are identical), we set $T_{cons}^{\left(CVM\right)}\leftarrow t$ and terminate. Otherwise we go back to step (i).

**Fig 2:**
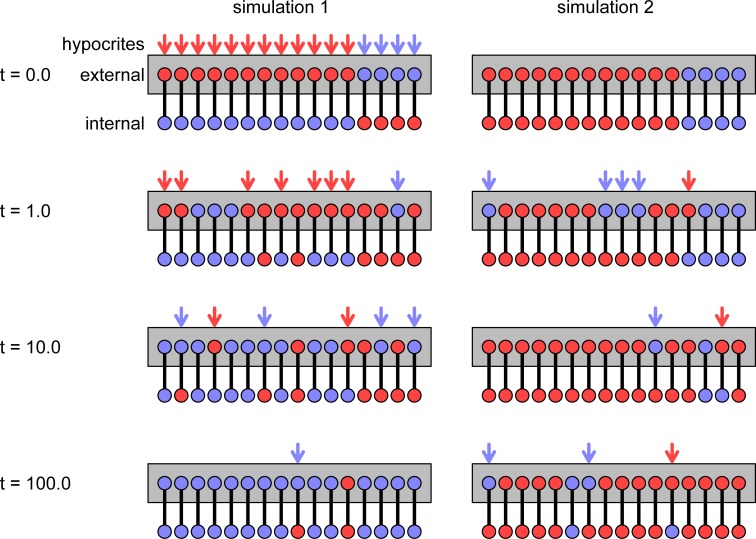
Two illustrative simulations in a relatively small group (*N* = 16 individuals). Each panel shows the state of the group at a given time from *t* = 0.0 to 100.0. (We write time with a decimal point because it is a continuous variable in our model.) The update rates are *c* = 1,*e* = 1/4, and *i* = 1/16. In each snapshot, the upper/lower nodes show the external/internal opinions (red or blue). In the external layer, the nodes are linked in a complete graph. We do not show the links, only indicate full connectedness by a gray box. (The reflexive self-link is also included. Therefore, every individual is linked with *N* = 16 individuals within the external layer.) The link between each individual’s external and internal opinion is represented by a vertical line. Arrows mark those individuals who are hypocrites. The color of the arrow shows the external opinion. Initially, both groups contain 12 *red and* 4 blue opinions in the external layer. The difference is in the occurrence of hypocrisy: in the left/right column, every individual is initially hypocritical/frank. By *t* = 100.0, one of the opinions has reached a significant majority in each simulation (i.e., the group is close to a consensus).

We show snapshots of two illustrative sample runs of the CVM algorithm in **Fig 2**.

The agent-based BVM and CVM algorithms that we have presented in this section make the relation between the models and individual opinion formation explicit. However, in practice, the corresponding Gillespie algorithms [69], based on the transition rate matrices given in [53], produce numeric results for both models more efficiently and are mathematically equivalent. We have, therefore, implemented our simulations with the Gillespie algorithms. The source code is available from GitHub [70].

## 3 Consensus time in the BVM and CVM

The BVM has been studied by numerous authors during its 45-year history (for reviews, see [12,14]), while the CVM is relatively new [53]. Here we review the main results concerning the consensus time in complete graphs. Both the BVM and the CVM lead to a consensus within a finite time, provided that the number of individuals *N* is finite (cf. [71] concerning the BVM and [53] about the CVM). The only exception occurs when *i* = 0 in the CVM and both *r* and *b* are present in the internal layer. In that special case, *r* and *b* persist for an infinitely long time.

In the BVM, the mean consensus time in the complete graph is [58]

$$T_{cons}^{\left(BVM\right)}(\rho_{R})=-\frac{N}{c}{[\rho }_{R}\;\ln\;\rho_{R}+(1-\rho_{R})\ln{(1-\rho }_{R})],$$ (1)

where *N* is the number of individuals, *c* is the copying rate, and *ρ*_*R*_ is the proportion of the red opinion in the external (i.e., only) layer at time *t* = 0. Note that $T_{cons}^{\left(BVM\right)}$ is symmetric under the exchange of the red and blue opinions because *ρ*_*B*_ = 1−*ρ*_*R*_. For obtaining the mean consensus time in the CVM, it is worth introducing a new variable,

$$m(\rho_{R},\rho_{r})=\frac{i\rho_{R}+e\rho_{r}}{e+i},$$ (2)

where *ρ*_*R*_ = *ρ*_*Rb*_+*ρ*_*Rr*_ is the fraction of *externally* red individuals and *ρ*_*r*_ = *ρ*_*Br*_+*ρ*_*Rr*_ is the fraction of *internally* red individuals (**Table 1**). We can interpret *m* as the overall “strength” of the red opinion because it characterizes the actual weight of the red opinion in the group, as *e* and *i* express the flow of information between the two layers. The expected value of *m* remains constant throughout the process of consensus formation; that is, *m*(*ρ*_*R*_,*ρ*_*r*_) is a martingale [53]. As a consequence, *m*(*ρ*_*R*_,*ρ*_*r*_) is equal to the probability that the consensus in a group with initial conditions (*ρ*_*R*_,*ρ*_*r*_) is red. (We will show numerical evidence for this equality in **Section 4.3**.)

The mean consensus time $T_{cons}^{\left(CVM\right)}$ in the CVM can be directly obtained from that in the BVM,

$$T_{cons}^{\left(CVM\right)}(m)=\tau (c,e,i)∙T_{cons}^{\left(BVM\right)}(m),$$ (3)

where

$$\tau (c,e,i)=\frac{(c+e+i){\left(e+i\right)}^{2}}{i[{\left(e+i\right)}^{2}+ci]}.$$ (4)

On one hand, because *τ*(*c*,*e*,*i*)>1, the mean consensus time is always longer in the CVM than in the BVM, given the same number of individuals *N*. Thus, the existence of hypocrisy in a group increases the consensus time. On the other hand, the probability *m* of a red consensus and the mean consensus time $T_{cons}^{\left(CVM\right)}$ are fully determined by *c*, *e*, *i*, *n*, *ρ*_*R*_, and *ρ*_*r*_ (see **Eqs** **2–4**), and thus do not depend on the amount of hypocrites *ρ*_*Rb*_ or *ρ*_*Br*_. To resolve this paradox, it is necessary to better understand the transient behavior of the CVM, particularly the role of hypocrites in the consensus-finding process. This observation motivated the present study.

## 4 Results

### 4.1 Consensus formation in the CVM

**Fig 3:**
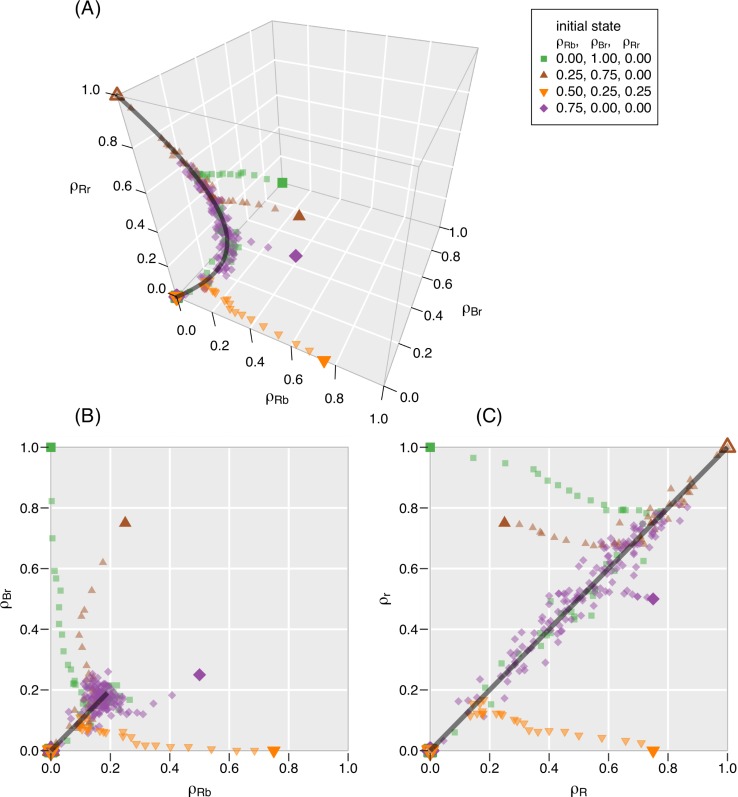
Changes in the composition of the group over time in four realizations of the CVM. Each point belongs to one “snapshot” between the initial state (large, filled symbols) and the consensus (large, open symbols). The parameters for all simulations are N = 400, c = 1, e = 1/4, i = 1/16. We show only a single simulation for each initial condition, but we note that repeated simulations from the same initial state can end in either of the absorbing states unless the initial state is already a consensus. The time intervals between the plotted points change along the trajectories: during the transient, we plot every 100^th^ time step until the 2000^th^ time step. Afterwards we plot only every 10000^th^ time step because the spatial density of data points is higher along the attractor. **(A)** Abundances of the two kinds of hypocrites (ρ_Rb_ and ρ_Br_) and the proportion of frank red individuals (ρ_Rr_). These variables completely describe the state of the system. The theoretical attractor **(Eq 5)** is shown as a black curve. **(B)** Equalization of the two kinds of hypocrites in the same realizations. In a consensus, there are no hypocrites (i.e., ρ_Rb_ = ρ_Br_ = 0). Therefore, the open symbols at the consensus overlap. The attractor appears as a straight line because it is a two-dimensional projection of the curve embedded in three dimensions. The line is exactly on the diagonal. **(C)** Equalization of the abundance of the red opinion in the external (ρ_R_ = ρ_Rb_+ρ_Rr_) and internal layer (ρ_r_ = ρ_Br_+ρ_Rr_). The attractor (black line) is exactly on the diagonal.

We study the changes in the state (*ρ*_*Rb*_,*ρ*_*Br*_,*ρ*_*Rr*_) over time. **Table 2** and **Fig 3** present results from simulations for four different initial conditions. All these simulations were performed with the same parameters *N* = 400, *c* = 1, *e* = 1/4, and *i* = 1/16. We have also carried out simulations for various other parameter combinations and report representative results in the S2 Appendix. In **Table 2**, we denote by *F* the fraction of simulations in which the consensus is red. (In the table, we also list the equilibration time *T*_equal_ that we define and discuss in **Section 4.2**.) **Table 2** underlines the excellent agreement between theoretical prediction and numeric simulations. We also observe agreement for other parameters. We support this claim by showing the results for another parameter combination in the S2 Appendix.

**Table 2:** The four cases of CVM simulations that are displayed in Figs 3 and 4.

Symbol & Color		■	▲	◆	▼
**Initial value**	***ρ***_***Rb***_	0.00	0.25	0.50	0.75
	***ρ***_***Br***_	1.00	0.75	0.25	0.00
	***ρ***_***Rr***_	0.00	0.00	0.25	0.00
***F***	**Observed**	0.81±0.02	0.65±0.03	0.57±0.03	0.17±0.02
	**Predicted** (*m*, see **Eq 2**)	0.80	0.65	0.55	0.15
$T_{cons}^{\left(CVM\right)}$	**Observed**	2630±160	3270±160	3470±150	2090±140
	**Predicted** (**Eq 3**)	2563.0	3316.2	3524.6	2165.1
***T***_**equal**_	**Observed**	3.23±0.05	3.23±0.11	3.14±0.23	3.15±0.07
	**Predicted** (**Eq 7**)	3.2			

The top row shows the symbols and colors used in the figures. The next three rows show the initial conditions. The remaining rows compare measurements from simulations (N = 400, c = 1, e = 1/4, i = 1/16) with theoretical values. Each measurement is the sample mean of 1000 simulations. Uncertainties are given as 95% confidence intervals. It is noteworthy that the time until consensus $T_{cons}^{\left(CVM\right)}$ is much longer than the equalization time T_equal_. (See **Section 4.2** for the definition of T_equal_.)

We show in **Fig 3A** and **3B** (and, for another parameter combination, in the S2 Appendix) that the process of consensus formation in all realizations takes place in two stages. The first stage is relatively short and is dominated by a directional change in the group’s composition: the trajectories go toward an attractor, which is an arch-shaped curve (plotted as a black curve in **Fig 3**). The S1 Appendix shows that the curve is given by

$$\rho_{Rb}=\rho_{Br}=\frac{\sqrt{{\left(e+i\right)}^{2}+4c(c+e+i)\rho_{Rr}}-(e+i)}{2c}-\rho_{Rr}.$$ (5)

The second stage of consensus formation is characterized by a random walk along this curve. The deviations of the trajectories from the theoretical curve are caused by the finite size of the simulated system (*N* = 400). The walk ends by reaching one of the two consensus states (i.e., *ρ*_*Rb*_ = *ρ*_*Br*_ = 0 and either *ρ*_*Rr*_ = 0 or *ρ*_*Rr*_ = 1). The direction of each step in the walk is randomly selected. However, unlike in conventional unbiased random walks, the frequency of steps is decreasing as the consensus is approached because most copying events are between like-minded individuals. Moreover, near the consensus, there are only few hypocrites left so that externalization and internalization are also unlikely to change the state.

It is interesting to observe the wide range of the walk in the second CVM stage. For example, the trajectory indicated by purple diamonds in **Fig 3C** ends in a consensus in which the blue opinion wins (i.e., *ρ*_*Rb*_ = *ρ*_*Br*_ = *ρ*_*Rr*_ = 0); nevertheless, the trajectory goes through some points in which *ρ*_*R*_>0.5, (i.e., the red opinion is temporarily the majority in the external layer). The probability of a reversal from an external red majority *ρ*_*R*_(*t*)>0.5 at time *t* to a blue consensus is equal to 1−*m*[*ρ*_*R*_(*t*),*ρ*_*r*_(*t*)], where *m* is given by **Eq 2**. We note that this probability does not depend on the system size because *m* is independent of *N*.

### 4.2 Equalization of the number of hypocrites

There are two kinds of hypocrites: *Rb* (i.e., externally red) and *Br* (externally blue). We denote the difference in the abundances by *D* = *ρ*_*Rb*_−*ρ*_*Br*_. In **Fig 3A**, we see that the abundances of the two kinds of hypocrites tend toward equality in the first stage of consensus formation (i.e., $\bar{D}\to 0$, where the overline denotes the mean, averaged over different simulations). A projection of the data onto the (*ρ*_*Rb*_,*ρ*_*Br*_) plane (**Fig 3B**) shows this tendency even more clearly. The black line (i.e., the projection of the attractor) is exactly on the diagonal.

**Fig 4:**
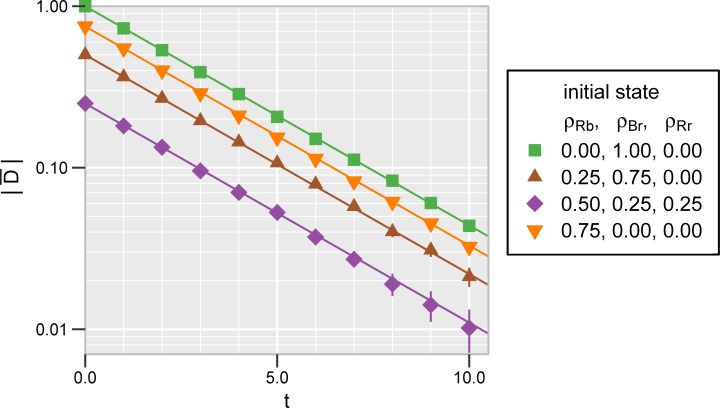
Equalization of the two kinds of hypocrites over time. The parameter values are the same as in **Fig 3**. The initial conditions are also the same for the corresponding shapes and colors. The vertical axis shows the absolute value of the mean difference $\bar{D}=\bar{\rho_{Rb}-\rho_{Br}}$ on a logarithmic scale. The mean was obtained from 1000 independent realizations. The error bars are shown only where they are larger than the symbol sizes. The solid lines are theoretical predictions under the assumption that $\bar{D}$ decays exponentially with rate e+i **(Eq 6)**.

To examine this phenomenon in more detail, we plot the absolute value of $\bar{D}(t)$ from *t* = 0.0 to 10.0 (**Fig 4**). The data points were obtained from 1000 independent repetitions. As we derive in the S1 Appendix, we expect

$$\bar{D}(t)=D_{0}∙exp[-(e+i)t].$$ (6)

(A rigorous proof is given in [53].) The straight lines in **Fig 4** indicate the theoretical predictions, which are an excellent fit to the numeric data.

In **Fig 3,** we illustrate that the equalization process is relatively fast compared to the subsequent random walk on the attractor. Let us define the equalization time *T*_equal_ as the time *t* satisfying $\bar{D}(t)=D_{0}∙exp(-1)$,

$$T_{equal}=\frac{1}{e+i}.$$ (7)

In the logarithmic plot of **Fig 4**, *T*_equal_ is proportional to the inverse of the slope. The plotted examples and the last two rows of **Table 2** confirm that *T*_equal_ is independent of the initial conditions. **Table 2** also shows that *T*_equal_ is much shorter than the mean consensus time $T_{cons}^{\left(CVM\right)}$. For the chosen rates (*c* = 1, *e* = 1/4, *i* = 1/16), $T_{cons}^{\left(CVM\right)}$ is longer than *T*_equal_ by around three orders of magnitude. The precise ratio of $T_{cons}^{\left(CVM\right)}$ to *T*_equal_ depends on the initial conditions and the parameters *c*, *e*, *i* and *N*. In particular, $T_{cons}^{\left(CVM\right)}$ increases linearly with *N* (see **Eqs** **1** and **3**), but *T*_equal_ is independent of *N*. So, even for only moderately large group sizes, the CVM spends most of the time during consensus formation in the second, random walk stage. Simulations with a different parameter combination (S2 Appendix) provide further evidence for the generality of this result.

The number of hypocrites is not the only observable that equalizes in the first stage. We show in **Fig 3C** that the abundance of the red opinion becomes equal in the public (external) and in the concealed (internal) layer (i.e., $\bar{\rho_{R}-\rho_{r}}\to 0$). The time of equalization is exactly the same as in the case of hypocrites above. The reason is that, because of *ρ*_*Rb*_ = *ρ*_*R*_−*ρ*_*Rr*_ and *ρ*_*Br*_ = *ρ*_*r*_−*ρ*_*Rr*_, we obtain

$$D=\rho_{Rb}-\rho_{Br}=\rho_{R}-\rho_{r}.$$ (8)

Consequently, *T*_equal_ applies as much to the equalization of the abundances between the two layers as it applies to the equalization of hypocrite types.

It is important to note that the ratio of red to blue is the same in both layers during the second stage, which is the predominantly longer stage during the process of consensus formation. This observation suggests that estimating the abundance of an opinion in the public layer gives a good prediction for the abundance in the concealed layer. (We consider the implications of this result in **Section 5.1**.) The statement refers, of course, to the *averages* in the population. The *individuals* who bear these opinions can be different at different times. Furthermore, **Eq 6** neither predicts *ρ*_*Rb*_ nor *ρ*_*Br*_, only the mean difference $\bar{D}=\bar{\rho_{Rb}-\rho_{Br}}$. At some parameter combinations, we temporarily find a considerable amount of hypocrites before reaching a consensus, while for other combinations most of the individuals are frank.

### 4.3 The (un)importance of hypocrisy

As we pointed out earlier, the opinion dynamics on a complete graph must end in either a red or blue consensus (provided that the number of individuals *N* is finite). How do the initial conditions and the rates *c*, *e* and *i* influence which opinion is going to win (i.e., becomes the consensus opinion)?

**Fig 5:**
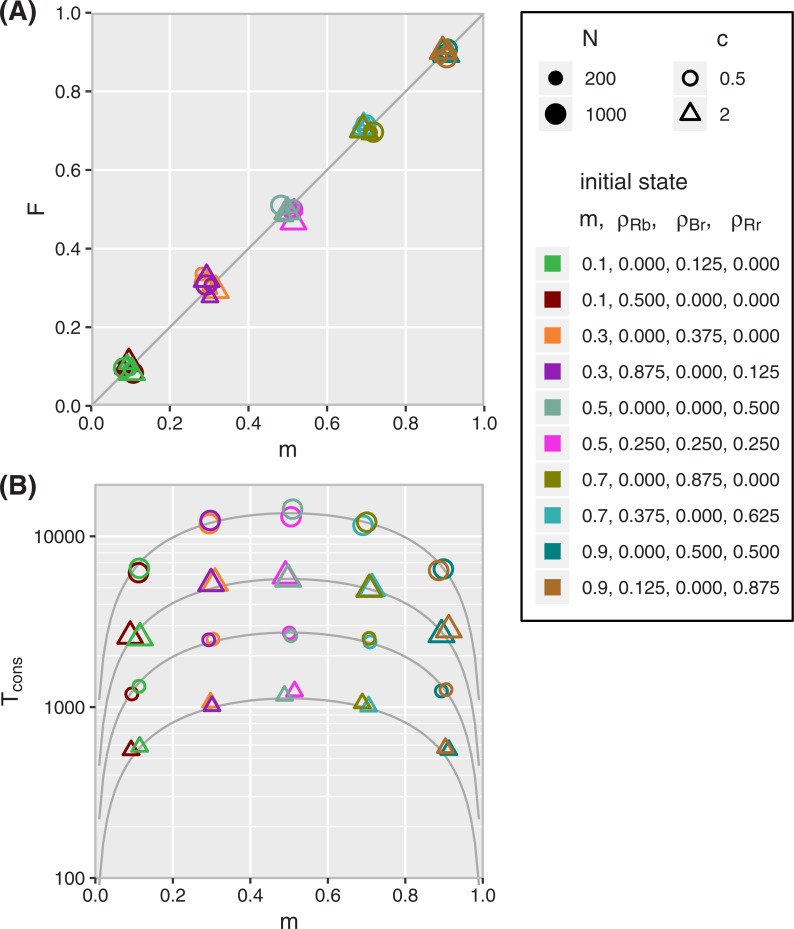
The consensus distribution and consensus time in the CVM. Numeric results confirm that these properties of the CVM depend only on m (defined in Eq 2), the initial strength of the red opinion. (In the plot, we have deliberately added jitter in the horizontal direction to make individual data points visible. Otherwise the overlap would obscure that there are multiple points on top of each other.) Each symbol shows the mean of 1000 simulations. All simulations use e = 1/4 and i = 1/16. N, c, and the initial conditions vary. (N is symbolized by the size, c by the shape, and the initial condition by the color; see legend.) **(A)** Proportion of simulations in which the red opinion wins (F) as a function of the initial strength of red [m(t = 0)]. The diagonal line indicates F = m. The overlapping symbols exemplify that simulations with different initial conditions produce the same F if the initial m is the same, despite different abundances of hypocrites. **(B)** The mean consensus time $T_{cons}^{\left(CVM\right)}$ as a function of m. The gray curves are theoretical predictions from **Eq 9**, which is derived under the assumption that N is large [53]. The simulations confirm that **Eq 9** is a good approximation even for moderately large N. For different N and c, the values of $T_{cons}^{\left(CVM\right)}$ fall on different curves. However, for given N and c, $T_{cons}^{\left(CVM\right)}$ depends only on m, but not on any further details of the initial conditions.

**Fig 5** shows theoretical predictions together with simulation results. We show in **Fig 5A** that the frequency *F* of red winning is equal to the initial value of *m*, defined in **Eq 2**. Since the formula for *m* does not contain *c*, the probability of winning does not depend on the communication at the external layer, but only on the interaction *between* the external and internal layer (expressed by *e* and *i*). Another noteworthy result is that the abundances of the hypocrites *ρ*_*Rb*_ and *ρ*_*Br*_ do not appear in **Eq 2** either. They are implicitly present in the equation because *ρ*_*R*_ = *ρ*_*Rb*_+*ρ*_*Rr*_ and *ρ*_*r*_ = *ρ*_*Br*_+*ρ*_*Rr*_. However, to predict the eventual winner, it is sufficient to know only two variables, namely *ρ*_*R*_ and *ρ*_*r*_. In fact, after the attractor (given by **Eq 5**; black curve in **Fig 3**) has been reached, we do not even need to know *ρ*_*r*_ any longer. By that time, the abundances in both layers are, on average, equal (see **Fig 3C**). So the probability of a red victory is directly given by the abundance *ρ*_*R*_ of red in the *external* layer, which is easier to observe in real life than the *internal* abundance *ρ*_*r*_.

Unlike *F*, the mean consensus time $T_{cons}^{\left(CVM\right)}$ does depend on *c* and *N*. This statement follows from the combination of **Eqs** **1**, **3** and **4**,

$$T_{cons}^{\left(CVM\right)}=-\frac{N(c+e+i){\left(e+i\right)}^{2}}{ci[{\left(e+i\right)}^{2}+ci]}[m\;ln(m)+(1-m)\;\ln\left(1-m\right)].$$ (9)

We confirm this result with **Fig 5B**: data points for different *N* and *c* fall on different curves. Still, neither *ρ*_*Rb*_ nor *ρ*_*Br*_ appear explicitly in **Eq 9**. Consequently, *m* is all we need to know about the initial condition in order to predict $T_{cons}^{\left(CVM\right)}$. After we have reached the attractor, *m* is equal to *ρ*_*R*_ so that we can, at that stage, make a prediction for the mean consensus time simply by observing the abundance of the red opinion in the external layer.

## 5 Discussion

### 5.1 The symmetry of hypocrisy

We studied the role of hypocrisy in a relatively simple model, the CVM in a complete graph. The CVM extends the original voter model (BVM) by incorporating an internal opinion that can be different from the external one (i.e., hypocrisy can occur).

In the CVM, each individual is exposed to two kinds of tension during the process of opinion formation,

- tension caused by the disagreement with another individual and
- cognitive dissonance [45] caused by a difference between the external and internal opinion of the individual.

Adopting the external opinion of a disagreeing neighbor releases tension (a). Externalization or internalization releases (b). In some cases, both types of tension can be simultaneously annihilated. In other cases, the reduction of one type of tension can directly lead to new tension of another type.

We showed that the consensus process in the CVM consists of two stages,

1. approaching the attractor (given by **Eq 5**) from the initial state and
2. a random walk on the attractor.

A characteristic feature of the attractor is *ρ*_*Rb*_ = *ρ*_*Br*_. That is, the hypocrites of both types (externally red and externally blue) are expected to be equally abundant, regardless of whether *R* or *B* is in a majority. For realistic parameters, stage 1 is much shorter than stage 2 so that *ρ*_*Rb*_≈*ρ*_*Br*_ for almost the entire duration of opinion formation. In particular, in the CVM even a great imbalance of opinions in the public layer (in our notation, a great difference between *ρ*_*R*_ and *ρ*_*B*_ = 1−*ρ*_*R*_) is accompanied by a balance of opinions among hypocrites (i.e., a small difference between *ρ*_*Rb*_ and *ρ*_*Br*_).

A second equality follows directly from the equalization of the hypocrites and **Eq 8**: $\bar{\rho_{R}-\rho_{r}}\to 0$. This means that the representation of the red opinion becomes the same in the external and the internal layer. This result might have some noteworthy consequences for opinion polls. Traditional survey methods sample the external layer only. In general, it is difficult to reach the internal layer (i.e., to get information about the concealed opinions) due to privacy or sensitivity concerns. In many cases, however, it would be crucial to know opinions hidden in the internal layer, especially when the action of the person is more strongly influenced by the internal than the external opinion. Examples include decisions whether to buy or consume a particular product or read an online source of information. Another example are elections, where the internal opinion is expressed by an action (e.g., voting for a party) without revealing the vote in public. Exit polls and surveys that mimic a secret ballot attempt to directly measure the internal layer.

The main consequence of our results is that a poll that asks only about the *external* opinions may still make a good estimate about the *internal* opinions as well, at least at the group level, assuming that there is no external desirability bias that would differentiate between the public expressions of the two opinions. Political polls are examples in which respondents can show a “public” face so that the internal opinion of many participants may remain hidden. This is even more likely in focus group studies or in a public discourse. If *ρ*_*R*_ = *ρ*_*r*_ holds, then this tendency does not make the poll invalid: the result of the poll may in fact be fairly accurate in spite of the occurrence of hypocritical answers. A remarkable result in the CVM is that neither the majority nor the minority opinions are externally overrepresented because of hypocrisy. In a neutral context without a media bias and an exogenous desirability bias, there is no expected difference between bandwagon and underdog hypocrisy.

## 5.2 How to shorten or prolong the consensus time

A long consensus time may be disadvantageous, for example, when a coordinated action is needed in a group. For instance, this is the case in jury decision making, which has been studied extensively [72–76]. Conversely, delaying a consensus may be desirable in those situations, in which the diversity of opinions is valuable. We have pointed out in **Section 4.2** that $T_{cons}^{\left(CVM\right)}$ increases linearly with *N* so that one strategy to delay a consensus simply is to expand the group size. The linear scaling of the consensus time with *N*, however, is not specific to the CVM. We find the same scaling relation in the BVM too (see **Eq 1**). Besides changing the group size, which properties of the concealed layer influence the consensus time?

We know from **Eq 4** that the delay *τ* depends only on the copying rate *c*, the externalization rate *e*, and the internalization rate *i*. If these rates are positive, we have *τ*>1 so that the CVM needs more time to reach consensus than the BVM with the same value of *c* [53]. For example, for the parameter combination used in **Table 2** (*c* = 1, *e* = 1/4, *i* = 1/16), we obtain *τ*≈12.8; that is, the CVM needs on average around one order of magnitude longer to reach a consensus. We are not aware of any empirical study that suggests specific values for *e* or *i*. To investigate the effect of varying *e* and *i*, we carry out simulations in the S2 Appendix where we swap their value compared to **Table 2** (i.e., *c* = 1, *e* = 1/16, *i* = 1/4). In that case, the consensus time is shorter, as predicted by **Eq 3**.

It is instructive to consider the limiting cases of the CVM if the rates *e* or *i* are extremely fast or slow. On one hand, in the limit *i*→∞ we would have simply retrieved the BVM. The prediction of **Eq 4** is, accordingly, *τ*→1 in this limit. On the other hand, increasing *e* and decreasing *i* can enhance the consensus time to enormous values. Thus, to maintain a longer coexistence of alternative opinions, one could aim to increase *e*, for example by encouraging self-expression [77–79], whereas decreasing *i* is more difficult to accomplish in real life because the institutions of socialization (school, work, the state, and the media) are well established. In the limit *i*→0, $T_{cons}^{\left(CVM\right)}$ goes to infinity. The reason for this divergence is that, in this case, the internal layer does not change at all so that any disagreement in the internal layer remains frozen in time.

The zealot voter model, introduced by Mobilia et al. [57], applies the limit *i*→0 together with *e*→∞ for a certain fraction of the group. These “zealots” never change their internal opinion and always express this opinion openly. Some other models in the literature can also be “translated” into two-layer voter models with fixed internal states (i.e., *i*→0). For example, there are models with “inflexible minorities” [80], “stubborn agents” [81], or “partisans” [59,60], in which the individuals have a fixed and innate preference for one of the (external) states. To the best of our knowledge, the CVM is the first model in which the internal layer can change so that the interaction between the two layers is bidirectional.

The winning opinion is not necessarily a majority opinion; for instance, as Nemeth and Wachtler [82] expressed, consistent minority views can affect other members of a jury whilst reaching their verdict in fictional courts. Minority opinions can also have an impact on public discourse in social questions such as feminism, the death penalty, or homosexuality [79,83,84].

The interplay between the layers slows down the CVM compared to the BVM, but, interestingly, the number of hypocrites is not directly responsible for the deceleration. As we pointed out above, the hypocrite abundances *ρ*_*Rb*_ and *ρ*_*Br*_ do not appear explicitly in the formulas for *m* and $T_{cons}^{\left(CVM\right)}$ (see **Eqs** **2** and **9**). In other words, both *m* and $T_{cons}^{\left(CVM\right)}$ are determined by the marginal frequencies in **Table 1** (i.e., *ρ*_*R*_ and *ρ*_*r*_) alone, which do not uniquely determine the joint frequencies *ρ*_*Rr*_, *ρ*_*Br*_, *ρ*_*Rb*_, and *ρ*_*Bb*_. For example, a group with *ρ*_*R*_ = 1/2 and *ρ*_*r*_ = 1/2 may consist purely of hypocrites (*ρ*_*Rb*_ = *ρ*_*Br*_ = 1/2, *ρ*_*Rr*_ = *ρ*_*Bb*_ = 0) or purely of frank individuals (*ρ*_*Rr*_ = *ρ*_*Bb*_ = 1/2, *ρ*_*Rb*_ = *ρ*_*Br*_ = 0). The consensus time is the same in both cases.

In this sense, the CVM is slower than the BVM because of the mere existence of the concealed layer and, thus, because of the *opportunity* for hypocrisy, but not because of the *amount* of hypocrisy. The concealed layer serves as a pool of opinions. Hypocrisy necessarily emerges in such a system as a path from one opinion to another, but the amount of hypocrisy does not drive the consensus process.

## 6 Summary and outlook

This study presented and analyzed the Concealed Voter Model (CVM) as a relatively simple model of opinion dynamics with the possibility of hypocrisy (i.e., internal and publicly expressed individual opinions might differ). The study demonstrated that the process of consensus formation in the model takes place in two stages. The first stage is relatively short and is characterized by an equilibration in the number of hypocrites of both types (*ρ*_*Rb*_≈*ρ*_*Br*_) or, equivalently, an equilibration in the opinions in both layers (*ρ*_*R*_≈*ρ*_*r*_). The second stage is a random walk along an attractor to which the opinion distributions quickly converge. We investigated the time $T_{cons}^{\left(CVM\right)}$ needed to reach a consensus and compared $T_{cons}^{\left(CVM\right)}$ to the consensus time $T_{cons}^{\left(BVM\right)}$ in the Basic Voter Model (BVM), in which hypocrisy is non-existent.

An important result is that hypocrisy always prolongs the mean time needed to reach unanimity of opinions. This result calls for policy measures that could eliminate or decrease the second stage of the dynamics in jury and committee decision making where consensus and fast decisions are highly desired, for instance by requiring a critical (high) threshold instead of unanimity [72,73].

As a second important result, the study showed that the number of both kinds of hypocrites equalize. This is good news for opinion pollsters who suffer from bias induced by hidden opinions. The model demonstrates that, in a neutral scenario, the numbers of both kinds of hypocrites balance each other out–so any empirical observation of bias is due to external factors or heterogeneous externalization and internalization rates that correlate with party preference.

As a third important result, the study demonstrated that in the CVM, just like in the BVM, the consensus time increases linearly with population size. This result is in line with observations that coordination in a larger group is more difficult, but not disproportionately more difficult than in a smaller group [72,74].

The simple CVM can be extended in multiple directions. So far, we have assumed that the links between individuals constitute a complete graph. It would be interesting to consider whether permitting different graph structures would change the results qualitatively. Previous work highlighted that the network structure is important for opinion dynamics in general [85]. In the BVM, networks with broad degree distributions shorten the time till consensus [58]. In a model similar to the BVM, Axelrod [86] has shown that minority opinions can survive and consensus may never be reached if network routes of influence are dissolved so that opinions are cut out completely from interactions with opposing opinions. Minority opinions are also likely to survive in models where network ties of influence are updated according to opinion homophily so that there is a co-evolution of networks and opinions [87–90].

Another way to increase realism is to consider nonbinary opinions. The previous literature has highlighted substantial differences between opinions measured on binary, continuous or nominal scales [91]. From an empirical perspective, it would also be desirable to study non-symmetric competition between the opinions. Asymmetry could be introduced, for instance, by a “media” effect that causes bias in one direction (e.g., in social influence or externalization) [92–94] or by heterogeneous internalization rates that correlate with voter preferences. Beyond a constant media bias in one direction, one could study exogenous stimuli that alter the direction of the bias. Such an extension could potentially explain the occurrence of cyclical patterns that are not emergent from the current model, but are observable, for example, in fashion trends and public sentiments [95,96].

Despite the limitations, we believe that studies that permit concealed opinions have a lot of potential in understanding opinion dynamics because they incorporate an important feature of real-life opinions: not everything is expressed openly.

## Supporting information

S1 Appendix Derivation of Eqs 5 and 6. (PDF)

S2 AppendixNumerical simulations and theoretical predictions for a different set of parameter values: *c* = 1, $e=\frac{1}{16},\;i=\frac{1}{4}$.(PDF)
