# Supplementary material for: The impact of hypocrisy on opinion formation: A dynamic model
Source: PLoS One. 2019 Jun 26;14(6):e0218729. doi: 10.1371/journal.pone.0218729 (PMC6594623; doi:10.1371/journal.pone.0218729)
Supplement: S1 Appendix — (PDF) [file pone.0218729.s001.pdf]

The impact of hypocrisy on opinion formation

Supporting information 1

Michael T. Gastner[1,2*], Károly Takács[2,3], Máté Gulyás[2,4], Zsuzsanna Szvetelszky[2], Beáta Oborny[4,5]

[1] Division of Science, Yale-NUS College, Singapore
[2] MTA TK "Lendület" Research Center for Educational and Network Studies (RECENS), Hungarian Academy of Sciences, Budapest, Hungary
[3] The Institute for Analytical Sociology (IAS), Linköping University, Norrköping, Sweden
[4] Department of Plant Taxonomy, Ecology and Theoretical Biology, Biological Institute, Loránd Eötvös University (ELTE), Budapest, Hungary
[5] GINOP Sustainable Ecosystems Group, Centre for Ecological Research, Hungarian Academy of Sciences, Tihany, Hungary

[*] michael.gastner@yale-nus.edu.sg

Appendix: derivation of equations 5 and 6

In the CVM, the state of the system can be characterized by the abundance of both types of hypocrites (ρ_{Rb} and ρ_{Br}) and red frank individuals (ρ_{Rr}). In one update, these variables change by at most $\pm 1/N$, where N is the number of individuals in the group. Suppose the previous state is $(\rho_{Rb}, \rho_{Br}, \rho_{Rr})$. There are eight possible events that cause a change from $(\rho_{Rb}, \rho_{Br}, \rho_{Rr})$. We list these events in Table S1.1 together with the corresponding transition rates. The rates are those of the CVM on a complete graph with self-links (i.e., we include a link from each node to itself). If we exclude the self-link, the transition rate c will have to be replaced by $c\frac{N}{N-1}$. Keeping the factor $\frac{N}{N-1}$ throughout the calculation would complicate the mathematical expressions without additional insight into the model. The "correction factor" $\frac{N}{N-1}$ is close to 1 even for moderately small groups and goes to zero in the limit $N \to \infty$.

We can express the dynamics of the expected values $\overline{\rho_{Rb}}$, $\overline{\rho_{Br}}$ and $\overline{\rho_{Rr}}$ by rate equations. For example, ρ_{Rb} increases by $\frac{1}{N}$ because of event 7 in Table S1.1, whereas events 1, 2, and 6 decrease ρ_{Rb} by the same amount. None of the other events change ρ_{Rb}. Taking the rates of the corresponding events into account, $\overline{\rho_{Rb}}$ evolves according to the differential equation

$$\frac{d\,\overline{\rho_{Rb}}}{dt} = c\,\overline{\rho_{Bb}}\,\overline{\rho_R} - e\,\overline{\rho_{Rb}} - i\,\overline{\rho_{Rb}} - c\,\overline{\rho_{Rb}}\,\overline{\rho_B}\ . \tag{S1.1}$$

Similar arguments show that

$$\frac{d\,\overline{\rho_{Br}}}{dt} = c\,\overline{\rho_{Rr}}\,\overline{\rho_B} - e\,\overline{\rho_{Br}} - i\,\overline{\rho_{Br}} - c\,\overline{\rho_{Br}}\,\overline{\rho_R} \tag{S1.2}$$

and

$$\frac{d\,\overline{\rho_{Rr}}}{dt} = i\,\overline{\rho_{Rb}} + e\,\overline{\rho_{Br}} + c\,\overline{\rho_{Br}}\,\overline{\rho_R} - c\,\overline{\rho_{Rr}}\,\overline{\rho_B}\ . \tag{S1.3}$$

Table S1.1. Transition rates in the CVM on a complete graph with self-links.

	Event	New state	Transition rate
1	An *Rb* individual externalizes	$\left(\rho_{Rb} - \frac{1}{N}, \rho_{Br}, \rho_{Rr}\right)$	$eN\rho_{Rb}$
2	An *Rb* individual internalizes	$\left(\rho_{Rb} - \frac{1}{N}, \rho_{Br}, \rho_{Rr} + \frac{1}{N}\right)$	$iN\rho_{Rb}$
3	A *Br* individual externalizes	$\left(\rho_{Rb}, \rho_{Br} - \frac{1}{N}, \rho_{Rr} + \frac{1}{N}\right)$	$eN\rho_{Br}$
4	A *Br* individual internalizes	$\left(\rho_{Rb}, \rho_{Br} - \frac{1}{N}, \rho_{Rr}\right)$	$iN\rho_{Br}$
5	An *Rr* individual copies a *B* individual	$\left(\rho_{Rb}, \rho_{Br} + \frac{1}{N}, \rho_{Rr} - \frac{1}{N}\right)$	$cN\rho_{Rr}\,\rho_B$
6	An *Rb* individual copies a *B* individual	$\left(\rho_{Rb} - \frac{1}{N}, \rho_{Br}, \rho_{Rr}\right)$	$cN\rho_{Rb}\,\rho_B$
7	A *Bb* individual copies an *R* individual	$\left(\rho_{Rb} + \frac{1}{N}, \rho_{Br}, \rho_{Rr}\right)$	$cN\rho_{Bb}\,\rho_R$
8	A *Br* individual copies an *R* individual	$\left(\rho_{Rb}, \rho_{Br} - \frac{1}{N}, \rho_{Rr} + \frac{1}{N}\right)$	$cN\rho_{Br}\,\rho_R$

We can express the right-hand sides of Eqs S1.1–S1.3 in terms of only ρ_{Rb}, ρ_{Br} and ρ_{Rr} by using the identities $\rho_R = \rho_{Rb} + \rho_{Rr}$, $\rho_B = 1 - \rho_{Rb} - \rho_{Rr}$ and $\rho_{Bb} = 1 - \rho_{Rb} - \rho_{Br} - \rho_{Rr}$. The result is the following set of equations:

$$\frac{d\,\overline{\rho_{Rb}}}{dt} = c\left(\overline{\rho_{Rb}} + \overline{\rho_{Rr}}\right)\left(1 - \overline{\rho_{Br}} - \overline{\rho_{Rr}}\right) - (c + e + i), \overline{\rho_{Rb}}, \tag{S1.4}$$

$$\frac{d\,\overline{\rho_{Br}}}{dt} = c\left(\overline{\rho_{Br}} + \overline{\rho_{Rr}}\right)\left(1 - \overline{\rho_{Rb}} - \overline{\rho_{Rr}}\right) - (c + e + i)\,\overline{\rho_{Br}}, \tag{S1.5}$$

$$\frac{d\,\overline{\rho_{Rr}}}{dt} = c\left(\overline{\rho_{Rb}} + \overline{\rho_{Rr}}\right)\left(\overline{\rho_{Br}} + \overline{\rho_{Rr}}\right) - c\overline{\rho_{Rr}} + e\overline{\rho_{Br}} + i\overline{\rho_{Rb}}. \tag{S1.6}$$

We obtain the evolution of the difference $\overline{D} = \overline{\rho_{Rb}} - \overline{\rho_{Br}}$ by subtracting Eq S1.5 from S1.4,

$$\frac{d\overline{D}}{dt} = -(e + i)\overline{D}, \tag{S1.7}$$

which explains Eq 6 in the main text. We can also infer the shape of the attractor (black curve in Fig 3) by setting the derivatives on the left-hand side of Eqs S1.4–S1.6 equal to zero. It follows that the attractor is given by Eq 5.
