# Supplementary material for: The impact of hypocrisy on opinion formation: A dynamic model
Source: PLoS One. 2019 Jun 26;14(6):e0218729. doi: 10.1371/journal.pone.0218729 (PMC6594623; doi:10.1371/journal.pone.0218729)
Supplement: S2 Appendix — (PDF) [file pone.0218729.s002.pdf]

The impact of hypocrisy on opinion formation

Supporting information 2

Michael T. Gastner[1,2*], Károly Takács[2,3], Máté Gulyás[2,4], Zsuzsanna Szvetelszky[2], Beáta Oborny[4,5]

1 Division of Science, Yale-NUS College, Singapore
2 MTA TK “Lendület” Research Center for Educational and Network Studies (RECENS), Hungarian Academy of Sciences, Budapest, Hungary
3 The Institute for Analytical Sociology (IAS), Linköping University, Norrköping, Sweden
4 Department of Plant Taxonomy, Ecology and Theoretical Biology, Biological Institute, Loránd Eötvös University (ELTE), Budapest, Hungary
5 GINOP Sustainable Ecosystems Group, Centre for Ecological Research, Hungarian Academy of Sciences, Tihany, Hungary

* michael.gastner@yale-nus.edu.sg

Appendix: numerical simulations and theoretical predictions for a different set of parameter values: $c = 1$, $e = 1/16$, $i = 1/4$

In the main text, we used $c = 1$, $e = 1/4$ and $i = 1/16$ for all simulations presented there. We have also carried out simulations for various other parameter choices and find generally good agreement between theoretical prediction and simulation. In this section, we show, as an example, results for a different set of parameter values: $c = 1$, $e = 1/16$, $i = 1/4$ (i.e., the values of e and i are interchanged).

Table S2.1 plays the equivalent role of Table 2 in the main text. Although for the new parameters, $T_{\text{cons}}^{(\text{CVM})}$ is shorter, it is still two orders of magnitude smaller than T_{equal} for all investigated initial conditions. Thus, the time until the external and internal proportions of red opinions are approximately equal is still much shorter than the time until consensus.

In Fig S2.1, we show the dynamics of the model with the new parameters, applying the same visualization method that we use in Fig 3 of the main text. From all initial conditions, the opinions

Table S2.1. The frequency F that red wins, the consensus time $T_{\text{cons}}^{(\text{CVM})}$ and the equalization time T_{equal} for $N = 400$, $c = 1$, $e = 1/16$, $i = 1/4$. Each measurement is the sample mean of 1000 simulations. Uncertainties are given as 95% confidence intervals.

		■	▲	▼	◆
Initial value	ρ_{Rb}	0.00	0.25	0.50	0.75
	ρ_{Br}	1.00	0.75	0.25	0.00
	ρ_{Rr}	0.00	0.00	0.25	0.00
F	Observed	0.20 ± 0.02	0.34 ± 0.03	0.70 ± 0.03	0.60 ± 0.03
	Predicted	0.20	0.35	0.70	0.60
$T_{\text{cons}}^{(\text{CVM})}$	Observed	309 ± 17	389 ± 19	361 ± 17	421 ± 19
	Predicted	295.2	381.9	360.3	397.0
T_{equal}	Observed	3.19 ± 0.04	3.19 ± 0.12	3.31 ± 0.21	3.23 ± 0.08
	Predicted	3.2			

quickly approach the attractor (black curve, given by Eq 5 in the main text). Note that we plot points much more frequently during the early phase of the model so that even only a few points near the attractor correspond to a long period spent on the attractor where $\rho_R \approx \rho_r$ (i.e., the proportion of opinions are approximately equal in the external and internal layer).

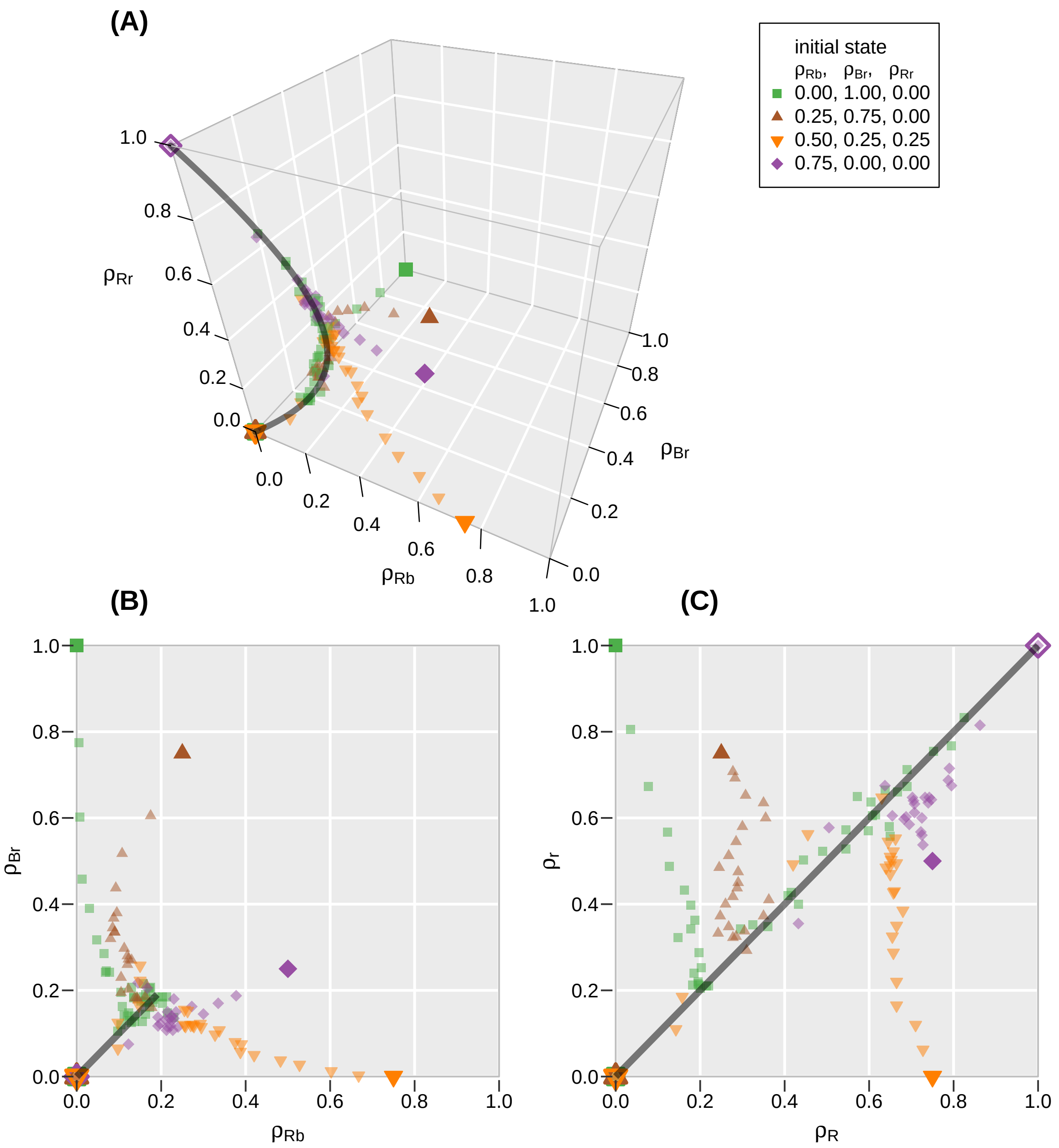

Fig S2.1. Changes in the composition of the group over time in four realizations of the CVM. In this figure, we use the same visualization method as in Fig 3 of the main text, but choose a different externalization rate ($e = 1/16$) and a different internalization rate ($i = 1/4$). As in the main text, the time intervals between the plotted points change along the trajectories: during the transient, we plot every 100th time step until the 2000th time step. Afterwards we plot only every 10000th time step. The attractor is shown as a black curve. **(A)** Abundances of the two kinds of hypocrites (ρ_{Rb} and ρ_{Br}) and the proportion of frank red individuals (ρ_{Rr}). **(B)** Equalization of the two kinds of hypocrites in the same realizations. **(C)** Equalization of the abundance of the red opinion in the external (ρ_R) and internal layer (ρ_r).
